# Solvent Screening for Solubility Enhancement of Theophylline in Neat, Binary and Ternary NADES Solvents: New Measurements and Ensemble Machine Learning

**DOI:** 10.3390/ijms22147347

**Published:** 2021-07-08

**Authors:** Piotr Cysewski, Tomasz Jeliński, Patryk Cymerman, Maciej Przybyłek

**Affiliations:** Department of Physical Chemistry, Faculty of Pharmacy, Collegium Medicum of Bydgoszcz, Nicolaus Copernicus University in Toruń, Kurpińskiego 5, 85-950 Bydgoszcz, Poland; tomasz.jelinski@cm.umk.pl (T.J.); patryk.cymerman@onet.pl (P.C.); m.przybylek@cm.umk.pl (M.P.)

**Keywords:** theophylline, solubility, machine learning, ensemble neural networks, COSMO-RS, NADES, binary solvents

## Abstract

Theophylline, a typical representative of active pharmaceutical ingredients, was selected to study the characteristics of experimental and theoretical solubility measured at 25 °C in a broad range of solvents, including neat, binary mixtures and ternary natural deep eutectics (NADES) prepared with choline chloride, polyols and water. There was a strong synergistic effect of organic solvents mixed with water, and among the experimentally studied binary systems, the one containing DMSO with water in unimolar proportions was found to be the most effective in theophylline dissolution. Likewise, for NADES, the addition of water (0.2 molar fraction) resulted in increased solubility compared to pure eutectics, with the highest solubilisation potential offered by the composition of choline chloride with glycerol. The ensemble of Statistica Automated Neural Networks (SANNs) developed using intermolecular interactions in pure systems has been found to be a very accurate model for solubility computations. This machine learning protocol was also applied as an extensive screening for potential solvents with higher solubility of theophylline. Such solvents were identified in all three subgroups, including neat solvents, binary mixtures and ternary NADES systems. Some methodological considerations of SANNs applications for future modelling were also provided. Although the developed protocol is focused exclusively on theophylline solubility, it also has general importance and can be used for the development of predictive models adequate for solvent screening of other compounds in a variety of systems. Formulation of such a model offers rational guidance for the selection of proper candidates as solubilisers in the designed solvents screening.

## 1. Introduction

Methylxanthines belong to the purine alkaloids and, as such, they comprise a fused heterocyclic system with pyrimidine and imidazole rings. Specifically, the studied compound, i.e., theophylline (T), is a bi-substituted derivative of xanthine (1,3-dimethylxanthine). Methylxanthines are abundant in nature and can be found in tea and other plant leaves, coffee and cocoa beans, as well as cola seeds [1]. Theophylline is mostly found in cocoa beans, with trace amounts in other sources [2]. It was chemically identified for the first time in 1888 and synthesised seven years later [1]. Methylxanthines play an important role in different biological processes, and their biological activities include the stimulation of the central nervous system, increased blood pressure, kidney diuresis, relaxation of smooth muscles, strengthening of the concentration of skeletal muscles and gastric acid secretion [3,4]. The mode of action of methylxanthines arises from their activities as phosphodiesterase inhibitors [5] and nonselective adenosine receptor antagonists [6]. Theophylline, in particular, is used to treat asthma, chronic obstructive pulmonary disease and neonatal apnoea [7,8,9,10,11,12]. It is usually administered in the form of a capsule or tablet and is extensively metabolised in the liver, with only a small part excreted in urine [13]. Theophylline has a low octanol-water partition coefficient (P) and is sparingly soluble in water [1].

The problem of the limited solubility of chemical compounds in water and various other solvents poses a challenge to many areas of modern industry, notably including pharmaceutics. Many substances are regarded as insoluble in aqueous solutions [14,15,16], which makes it necessary to develop techniques that would overcome this difficulty. Having the above in mind, several different approaches have been offered to address poor solubility. Among the most often utilized are micronization [17], monocrystal formation [18,19], amorphization [20,21,22], complexation with cyclodextrins [23,24], solid dispersion formation [25], pH modification [26,27], formation of salts [28,29] and cocrystals [30,31]. The usage of hydrotropes or cosolvation techniques also offers an interesting and relatively simple way of enhancing solubility [32,33]. The cosolvation effect takes place when a particular amount of a cosolvent is added to the primary solvent and causes a solubility increase [34]. Among the organic compounds primarily used for this task, one can include alcohols, DMSO, propylene glycol or glycerin [35,36]. However, the usage of organic solvents may have limited applicability due to their potential toxicity. This generates a need for alternative solvation media that would combine good solubilising potential with environmental and health safety. These simultaneous requirements focus the attention of many researchers on natural deep eutectic solvents (NADES). The key feature of deep eutectic solvents (DES) is the lowering of their melting point in comparison with the corresponding individual components. This enables them to form liquids even at room temperature [37]. Natural deep eutectic solvents are a specific part of this larger group of compounds and can be defined as bio-based DES prepared using such compounds as organic acids, alcohols, amino acids, sugars, choline chloride (ChCl) or other plant-based primary metabolites [38,39]. The desired physicochemical properties of NADES, including low volatility, sustainability and biodegradability, low cost and simplicity of preparation, as well as the potential of fine-tuning for specific applications, make these solvents very favourable in terms of efficiency, safety and economics [40,41,42,43]. All of this leads to the widespread usage of natural deep eutectic solvents in many different applications [42,44,45,46,47,48,49].

During the process of extraction, synthesis, evaluation and optimisation of active pharmaceutical ingredients (APIs), a large volume of solubility data is required, both in neat solvents as well as their mixtures [36]. For example, when considering cosolvation, it would be beneficial to know which potential cosolvents are the most promising before conducting actual experiments. These considerations mean that a large number of experiments are needed to supply these data, which in turn results in a large amount of waste originating from a number of processes, including synthesis, purification and analysis. Therefore, it would be beneficial from an economical, ecological and environmental perspective to utilise such methods that would provide the necessary data but would simultaneously reduce the amount of environmental pollution. The answer to this problem might be the application of computational methods that would direct and partially replace the actual experiments. Unfortunately, to date, there are few reliable models enabling the estimation of solubility from the molecular structure of API and solvent molecules. Although many theoretical approaches were proposed [35,50,51,52], their usage is seriously prohibited by inadequate accuracy or restricted applicability domains resulting from limited training sets. That is why post-factum models, which use some equations with parameters fitted to experimental data, are so popular [53,54,55,56,57]. Furthermore, several alternative approaches were proposed to utilise physicochemical properties as molecular descriptors; for example, the general solubility equation proposed by Jain and Yalkowsky [58] utilises values of the melting point and the octanol-water partition coefficient. Moreover, the set of models developed by Abraham and coworkers [59] is worth mentioning; this type of approach allows for predicting the relative solubility with respect to a selected reference solvent, which is typically water. The set of equations proved to be very effective in solubility computations not only in organic solvents [60,61] but also in ionic liquids [62] after parametrisation with the aid of linear free energy relationships. On the other hand, ab initio approaches were developed for a priori characteristics of chemical systems without external parametrisation. Among them, one of the outstanding is the COSMO-RS model [63,64], extensively utilised for predicting solubility [65,66], solvent screening [67,68,69] and multiphase chemical equilibria [70,71,72] such as SLE, LLE, VLE, SLLE, etc. It combines density functional theory calculations with statistical thermodynamics as a post-treatment [73] and has become a standard approach in many scientific and industrial applications. Furthermore, dissolution in NADES [74,75] was studied using this theoretical framework. Unfortunately, the accuracy of this approach is very often not sufficient, not only for reproducing experiment data but also for screening purposes. That is why the advantages of machine learning [76] in general and the artificial neural networks (ANNs) [77] particularly have become a very powerful tool for solubility predictions proving their efficiency in many applications [78,79,80,81,82]. Although such non-linear modelling sacrifices the analytical solution, it can address serious accuracy problems, as previously documented [83]. That is why the effort was undertaken to effectively combine the machine learning protocol with molecular descriptors characterising properties of the solute in a variety of solvents for a quantitative API solubility computation. Although here the solubility of theophylline is addressed, the proposed procedure is general and can be easily applied to any API in virtually any solvent, including screening of new hydrotropic and green solvents. There are three main features of the protocol applied here. The starting point was the collection of available experimental solubility data of theophylline in the literature, which were seriously extended with new measurements. It is worth emphasising that quite a unique set of data was composed. It encompasses not only the values characterising solubility in neat solvents and five aqueous binary mixtures with organic liquids but also a set of NADES containing choline chloride mixed with several other compounds. In the second stage, the machine learning protocol was used for the development and validation of an ensemble of artificial neural networks. Finally, extensive screening was performed for finding the most promising solvents in all three categories, which led to proposing new solubility enhancers.

## 2. Results and Discussion

The screening protocol for solvents with enhanced solubility was divided into three steps. First, the data set of theophylline solubility was prepared by the collection of published data and seriously extended with new measurements. Then, using machine learning protocols, the non-linear model was developed by formulation and validation of an ensemble of artificial neural networks. Finally, extensive screening was performed for finding new promising solvents in all three categories as potential solubility enhancers.

### 2.1. Theophylline Solubility

**Figure 1:**
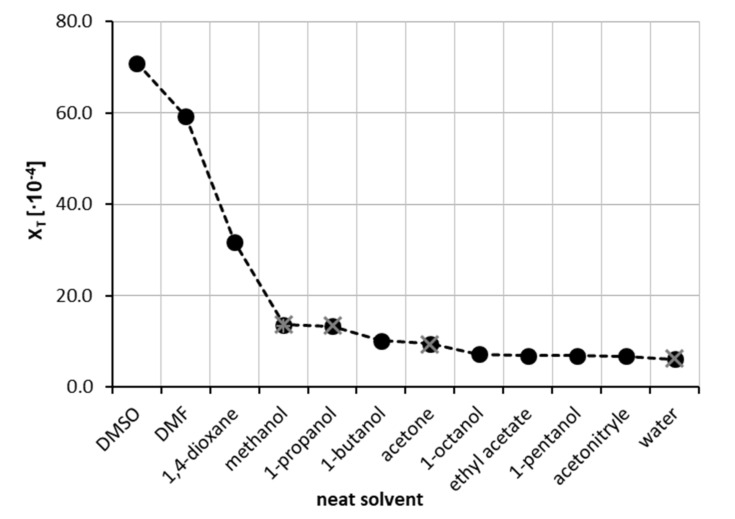
Solubility of theophylline at 25 °C, expressed as its mole fraction, x_T_, in eleven neat organic solvents and water. Black circles represent measurements of this work, while grey crosses indicate values published by Zhong et al. [84].

The starting point was the experimental determination of theophylline solubility in eleven neat organic solvents at room temperature. This enabled the initial solvent screening and preselection for the binary mixture solubility measurements. The resulting solubilities expressed as mole fraction x_T_, that were collected are shown in Figure 1. It happened that the highest solubilities were found for DMSO (x_T_ = 70.96 ± 0.28 × 10^−4^) and DMF (x_T_ = 59.21 ± 0.43 × 10^−4^). A slightly lower solubility of theophylline was observed for neat 1,4-dioxane (x_T_ = 31.67 ± 0.58 × 10^−4^), with other most effective solvents being methanol, 1-propanol and 1-butanol (x_T_ = 13.64 ± 0.04 × 10^−4^, 13.39 ± 0.13 × 10^−4^ and 10.23 ± 0.11 × 10^−4^, respectively). On the other hand, as expected, the smallest solubility was obtained for pure water (x_T_ = 6.10 ± 0.12 × 10^−4^) at ambient conditions. The five most effective solvents were selected for the next stage of experiments aiming for the determination of solubility in aqueous binary mixtures. It is worth mentioning that some of our measurements intentionally repeated the already available solubility data in four neat solvents. This allowed for direct comparisons of the accuracy of the performed experiments with published data. As demonstrated in Figure 1, our results are consistent with the previously determined solubility of theophylline by Zhong et al. [84]. A detailed comparison of our measurements with already published data is provided in the Supplementary Materials (see Table S2). It was found that the measurements presented in this work are in good accordance with the literature data, with a mean relative difference as low as 1.9%.

**Figure 2:**
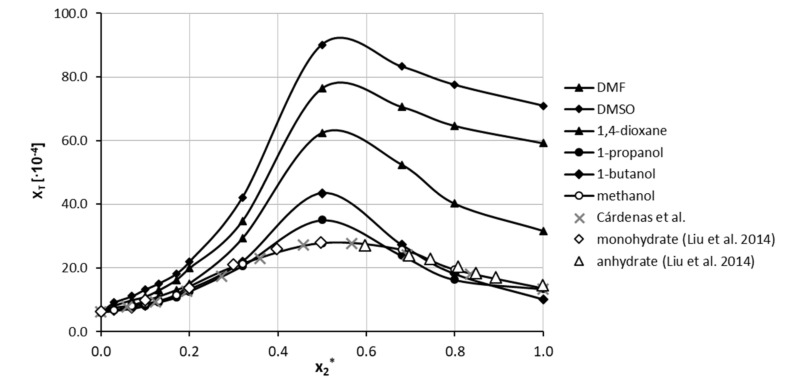
Solubility of theophylline at 25 °C, expressed as its mole fraction, x_T_, in binary solvents comprising water and five different organic solvents in varying compositions. On the ordinate, $x_{2}^{*}$ represents the mole fractions of organic solvent in solute-free binary solutions. The values of Theophylline solubility in aqueous methanolic solutions published by Cárdenas et al. [85] were marked with grey crosses and are anhydrous. Additionally, monohydrate and anhydrate theophylline solubilities measured by Liu et al. [86] were plotted as open diamonds and triangles.

In the next step, the solubility of theophylline was determined in five binary mixtures comprising DMSO, DMF, 1,4-dioxane, 1-propanol and 1-butanol mixed with water in twelve molar ratios covering the whole range of concentrations. The results of these measurements are presented in Figure 2. The most interesting observation is that all of the studied organic solvents exhibit synergistic behaviour since the highest solubility was obtained when the binary mixture comprised water and solvent in unimolar proportions. Likewise, all of the considered solvents can be regarded as good cosolvents since a systematic and significant solubility increase is observed with the rise of the organic solvent content in water solutions. The highest solubility at the point corresponding to $x_{2}^{*}$ = 0.5 was found to be equal to x_T_ = 90.13 ± 0.56 × 10^−4^ and x_T_ = 76.41 ± 0.58 × 10^−4^ for DMSO and DMF, respectively. In the case of 1,4-dioxane, the synergistic effect was slightly less pronounced, and the largest solubility was found to be equal to x_T_ = 62.37 ± 0.45 × 10^−4^. For the last two solvents, namely 1-butanol and 1-propanol, the largest solubility at unimolar solvent composition was found to be equal to x_T_ = 43.47 ± 0.22 × 10^−4^ and x_T_ = 35.03 ± 0.08 × 10^−4^, respectively. Interestingly, although the solubility of theophylline in neat 1-propanol was larger than in 1-butanol, for unimolar compositions with water, the order is reversed. Detailed results of theophylline solubility in both neat organic solvents and binary mixtures with water were collected in Tables S3 and S4 in Supplementary Materials.

**Figure 3:**
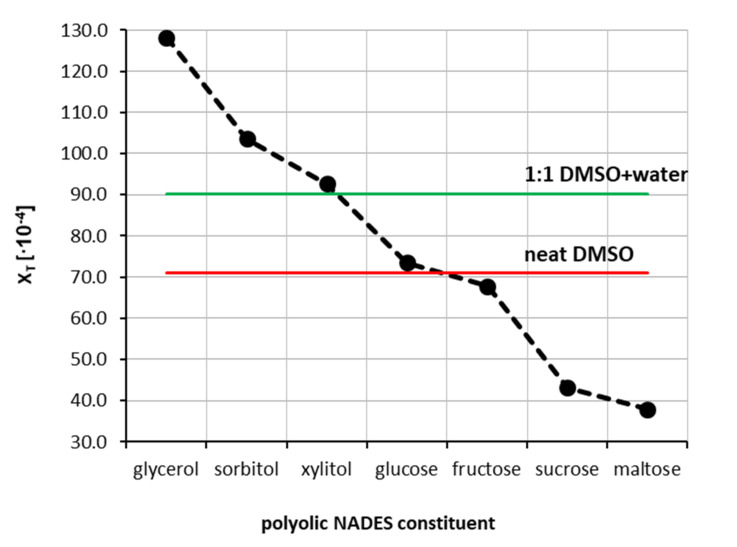
Mole fraction solubility of theophylline determined in water-free natural deep eutectic solvents at 25 °C. All designed solvents were prepared in unimolar proportions.

**Figure 4:**
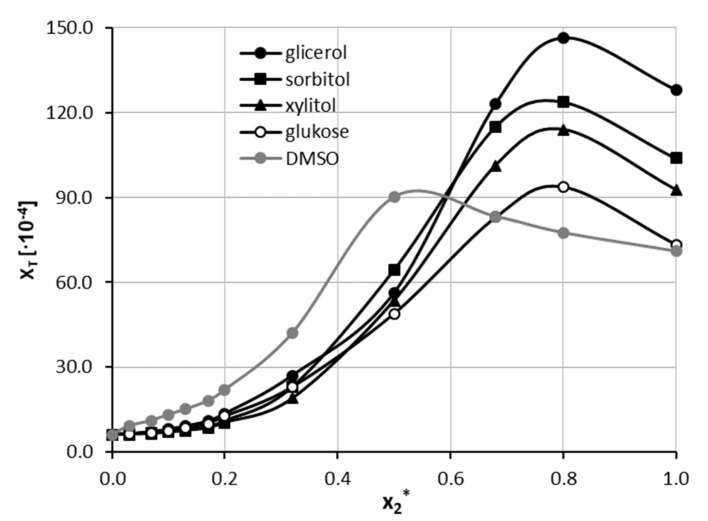
Solubility profiles of theophylline at 25 °C in mixtures comprising NADES successfully diluted with water. On the ordinate, $x_{\mathrm{NADES}}^{*}$, represents values of the mole fractions of Natural Deep Eutectic Solvent in aqueous solutions. For comparison purposes, solubility in DMSO+water was plotted as a function of $x_{2}^{*}$ (grey line).

The third series of measurements was devoted to the extension of the experimental pool of theophylline solubility by including some designed solvents. Hence, seven natural deep eutectic solvents containing choline chloride and one of the selected polyols were prepared by mixing both the cationic and anionic counterparts in unimolar proportions. This led to the identification of the NADES constituents, which are the most suited as potential theophylline solubility enhancers. The obtained results are presented in Figure 3. The highest solubility of theophylline was found in the NADES made of choline chloride and glycerol. The measured mole fraction in saturated solution was equal to x_T_ = 128.17 ± 0.68 × 10^−4^ and enhanced solubility 21 times compared to pure water and was about 80% higher with respect to pure DMSO. This observation provides direct evidence of the effectiveness of NADES as a proficient solubiliser of theophylline. Other polyols such as sorbitol and xylitol were not as efficient, resulting in lower theophylline solubility, and were equal to x_T_ = 103.63 ± 1.20 × 10^−4^ and x_T_ = 92.61 ± 0.94 × 10^−4^, respectively. NADES made of glucose, and fructose resulted in slightly lower solubility of theophylline, i.e., x_T_ = 73.45 ± 0.75 × 10^−4^ and x_T_ = 67.79 ± 0.72 × 10^−4^, respectively. It is worth noting that NADES formulated with the first four constituents gave solubility values of theophylline greater than in DMSO, which was characterised by the highest solubility among studied aqueous organic solvents. Taking this into account, the four most effective NADES were selected as cosolvents in mixtures with water, analogically to the earlier procedure with neat organic solvents. Hence, NADES with kept proportions of cationic and anionic counterparts were successfully diluted with water in the whole range of concentrations. This procedure allowed for determining the effectiveness of NADES as cosolvents for theophylline solubilisation, which was summarised in Figure 4. Additionally, the synergistic effect was observed, corresponding to the mixtures containing $x_{2}^{*}$ = 0.8 of designed solvents. The order of increased solubility observed for pure NADES solutions was also preserved in all studied mixtures with water. In the case of NADES comprising choline chloride and glycerol, the highest solubility of theophylline was found to be x_T_ = 146.57 ± 0.72 × 10^−4^, which stands for 24 times higher solubility with respect to pure water and 62% higher than the most effective binary mixture. When sorbitol and xylitol were used as the second NADES constituent, the resulting solubility was equal x_T_ = 123.83 ± 0.70 × 10^−4^ and x_T_ = 113.94 ± 0.31 × 10^−4^, respectively. Finally, for the NADES made with glucose, the solubility in the optimal composition was x_T_ = 93.76 ± 0.96 × 10^−4^. When comparing pure NADES and NADES-mixtures with water, the increase in theophylline solubility in the most effective composition is less pronounced than it was for the binary solvents tested earlier, although the solubility values are still greater than for DMSO. For the NADES with sorbitol, xylitol and glucose as the second constituent, the solubility of theophylline in the NADES-water mixture with $x_{2}^{*}$ = 0.8 is also larger than in pure NADES. It is thus expected that a modest amount of water is a promoting factor of theophylline solubility in organic solutions. Detailed results of theophylline solubility in both pure NADES and NADES-water mixtures can be found in Tables S5 and S6 in Supplementary Materials.

### 2.2. Precipitate Characteristics

Theophylline belongs to a class of organic solutes that exhibit rich structural diversity in the solid-state. The commercial theophylline is usually offered in the form of polymorph II since it easily crystallises from many neat organic solvents [87,88,89], and for a long time, it was considered to be the most stable form thermodynamically at room temperature [87,90,91]. However, it was documented by Seton et al. [89] that form IV is more stable at ambient conditions, although the II → IV conversion encounters a kinetic barrier significantly slowing the spontaneous transformation [89]. Form I is stable at higher temperatures [87], and form III is highly metastable at any temperature [92]. Hence, it is expected that the precipitate of theophylline from solutions under the solubility measurement conditions adopts the form II. However, theophylline can also exist as a stable monohydrate at conditions of sufficiently high water activity in the crystallisation medium [93]. For example, a spontaneous transformation of theophylline anhydrate into monohydrate occurs in contact with an aqueous methanolic solution if the water activity exceeds 0.252 [93]. On the contrary, anhydrous theophylline remained unchanged at conditions with a water activity below 0.227. This was also studied in detail [94] by precise determination of the transformation point of solution mediated transformation of monohydrate to anhydrate in form II. 

**Figure 5:**
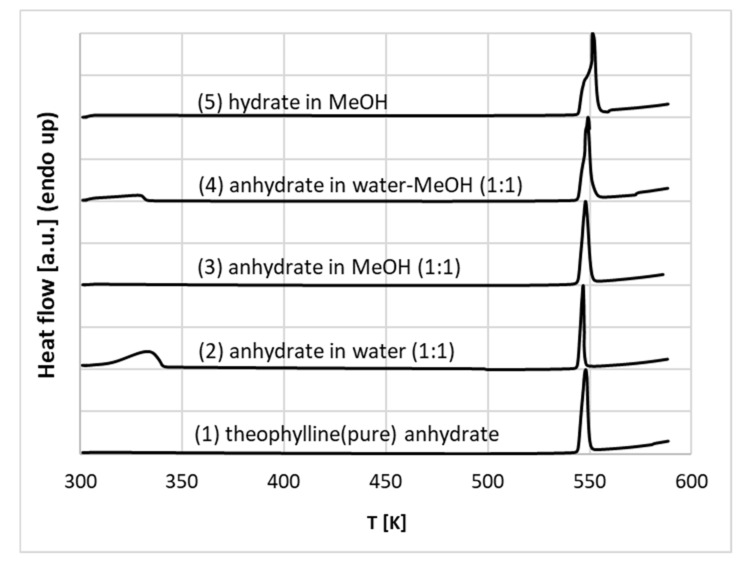
Thermograms of the products of milling of pure commercial anhydrated theophylline (1), anhydrate co-grinded with equimolar amounts of neat water (2), neat methanol (3), aqueous unimolar mixture with methanol (4) and monohydrate co-grinded with methanol (5).

To further explore this aspect, the mechanochemical synthesis was used for theophylline wetted with pure water, methanol and aqueous methanolic mixtures. The resulting thermograms of co-grinded samples provided in Figure 5 confirm the existence of a hydrate in water-rich systems since a characteristic small peak appeared at the low-temperature values. This endothermic process is typical for the theophylline-water complex [95,96]. As one can see, the commercial theophylline and the same compound in the presence of pure methanol adopt an anhydrous form, as clearly evidenced by the lack of peak around 340 K on lines (1) and (3) plotted in Figure 5. However, milling in the presence of pure water and aqueous methanol mixtures with a modest concentration of organic solvent results in the formation of a monohydrate with a characteristic peak on lines (2) and (4). Similar conclusions were also drawn from milling in neat water by González et al. [97]. Here, additional experiments were conducted by re-milling the freshly obtained hydrate in conditions of higher content of methanol. This resulted in the disappearance of the hydrate, as evidenced by line (5) in Figure 5. This is in good accord with the quantitative characteristics of aqueous methanol binary mixtures made by Liu et al. [86]. This observation serves as a general characteristic of theophylline behaviour in saturated solutions. Hence, the conditions of the solubility measurements determine the form of theophylline precipitate since the monohydrate-anhydrate transition is energetically reversible [94].

It is worth emphasising that despite the fact that in the case of theophylline, different precipitates occur with varying binary mixture compositions, the same set of parameters were used for modelling the solubility in the whole range of solvent ratios. For example, CNIBS/R-K and the modified Jouyban−Acree models were applied for interpreting the solubility of theophylline in methanol-water and isopropanol-water conditions [86]. Moreover, rich solid-liquid phase diagrams of theophylline were computed using the same set of parameters in PC-SAFT modelling [90]. Besides, preferential solvation of theophylline in methanol-water mixtures was characterised using the same idea [85]. This can be justified by the fact that the physicochemical properties of dissolved theophylline depend only on the bulk liquid in an equilibrated state, and the form of the solid-state does not interfere with the molecular parameters of the dissolved solute. The same approach was adopted here in formulating machine learning models since all parameters used for training characterise theophylline in the dissolved state. Hence, the solubility predictions can inform the bulk concentration but not about the form of the precipitate. Although this restriction is important for any model of solubility computations, it does not prohibit its applications for back solubility computations or screening purposes.

### 2.3. Predictive Solubility Model

**Figure 6:**
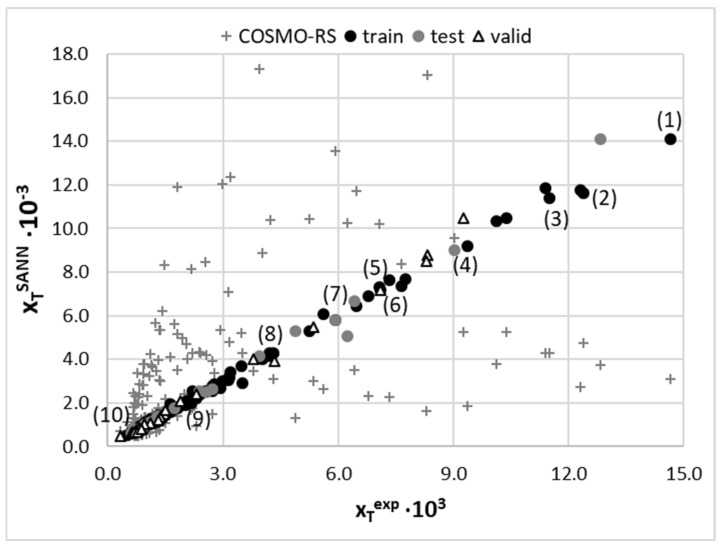

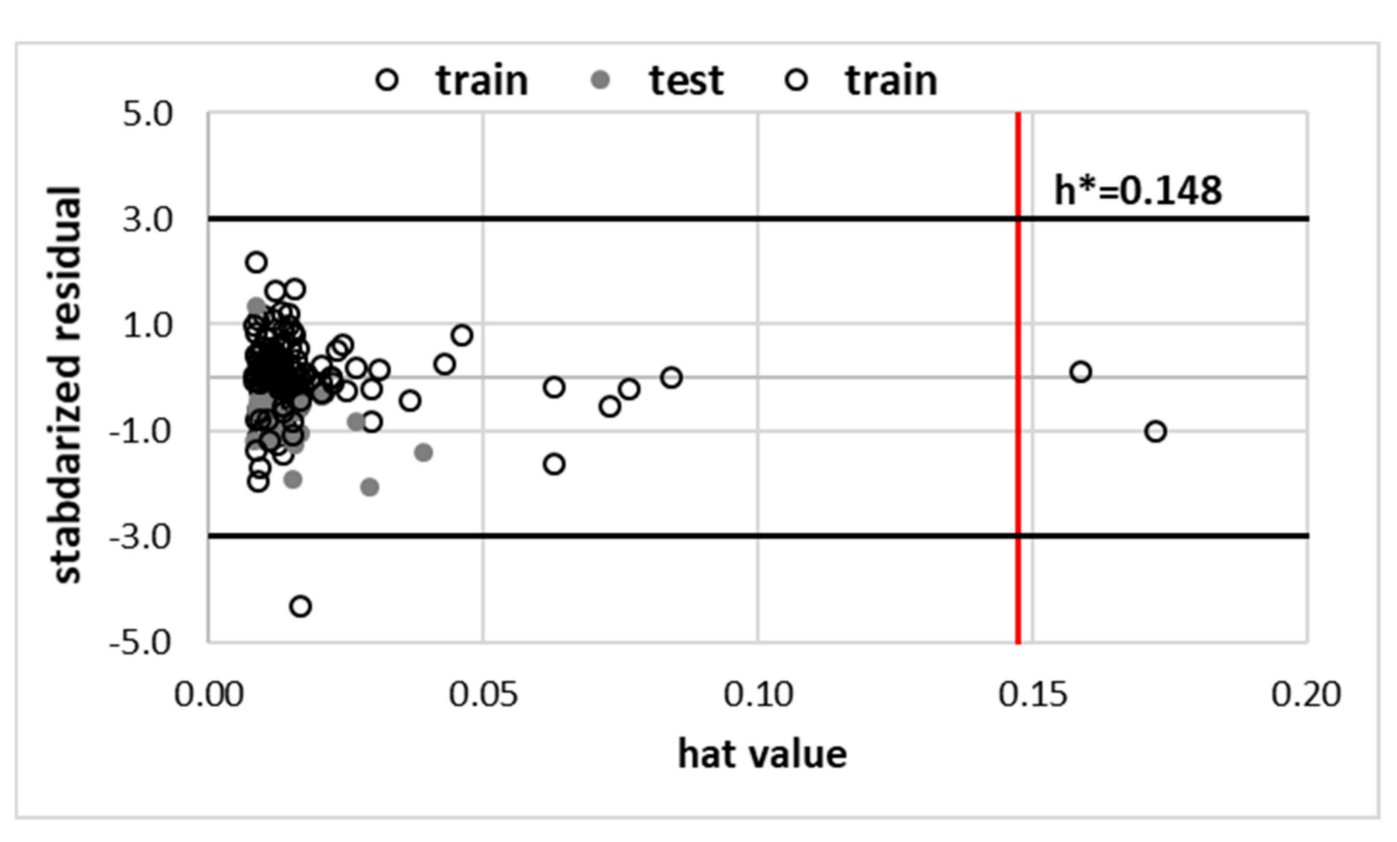
Accuracy of solubility prediction using COSMO-RS and an ensemble of SANNs, (top) relationship between experimental and predicted values of theophylline solubility in studied solvents and (bottom) applicability domain of a SANNs ensemble defined by relationship between standardized residuals and hat values. Some selected solvents were marked as (1) [ChCl+Glycerol+water] (0.8), (2) [ChCl+Sorbitol+water] (0.8), (3) [ChCl+Xylitol+water] (0.8), (4) [DMSO+water] (0.5), (5) [DMF+water] (0.5), (6) [DMSO], (7) [1,4-dioxane+water] 0.5, (8) 1-propoanol (9) acetone and (10) water.

The set of experimentally derived data comprising 160 measurements was used for the theoretical dissemination. In the first stage, the first principle approach utilising COSMO-RS theory was applied for direct solubility predictions. Unfortunately, it is clear that this approach is hardly suited for solubility predictions of theophylline in the studied solvents, as documented in Figure 6. The average percentage error exceeds 103%, and such high inaccuracy prevents the utilisation of these computations as a screening tool and guidance for the rational selection of solvents or their mixtures for enhancing solubility. The error introduced by COSMO-RS is of a not-systematic type, and no linear relationship was found for the improvement of the computed solubility. That is why an alternative approach was adopted, taking advantage of the non-linear machine learning protocol described in the methodology. The predictive power of any machine learning procedure relies on an adequate selection of molecular descriptors. The first choice of such parameters, very often utilised in modelling, comes from well-established [98,99,100] sets of 2D or 3D descriptors. Such an approach was successfully demonstrated in the case of co-crystal screening [101] or screening for solid curcumin formulations [102]. Many alternative molecular descriptors are of potential interest, as reviewed by Bergström and Larsson [103]. However, the application of such datasets for studies of temperature-dependent properties and/or multinary systems is problematic. Here, an alternative source of molecular descriptors was proposed. According to common chemical intuition, the concentration of saturated solution depends strongly on the intermolecular interactions. Hence, it seems plausible to utilise the energetic contributions as they can vary with the composition and measurements conditions. The first principle computations offer direct insight into the amount of hydrogen bonding energetics along with quantified dispersion and electrostatics. These data were taken from routine COSMO-RS computations, as described in the methodology. Although the solubility computed using COMSOtherm software suffers from serious inaccuracies, the energetic contributions provide sufficiently detailed information about chemical systems. It happened that the set of seven descriptors characterised the whole chemical space accurately enough to allow for the precise prediction of solubility in such a diverse set of solvents, including neat solvents, their binary mixtures and ternary NADES formulations. Since solubility values computed using COSMO-RS methodology have been found to be highly inaccurate, their values were not used as molecular descriptors, and only intermolecular interactions were included. The accuracy of the obtained model, along with its applicability domain, was presented in Figure 6. The mean RMSD value and the one characterising the best SANN were 36.6 × 10^−3^ and 29.3 × 10^−3^, respectively. The ensemble of neural networks comprises one-layer networks with 6 to 12 neurons. The detailed characteristics of the developed ensemble are provided in Supplementary Materials, Table S7. Since one of the inclusion criteria was the number of outliers not exceeding four, all SANNs are well suited for the theophylline solubility reproduction. The most common outliers in many networks were the cases of cold water solubility (T = 14.5 °C) and half diluted solvents [ChCl+Glycerol], i.e., NADES mixed with unimolar proportions with water.

### 2.4. Theophylline Solubility Screening

**Figure 7:**
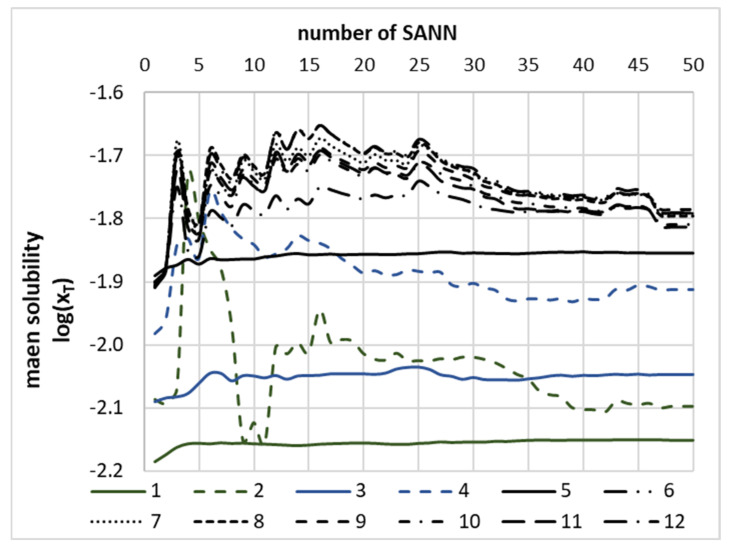
The distribution of mean values of theophylline solubility, expressed as the logarithm of mole fraction in the selected solvents, as a function of the number of neural networks taken for averaging. All accepted SANNs were sorted with decreasing accuracy. Legend caption: neat solvents: 1: neat DMSO, 2: neat 1-methyl-2-pyrrolidone, aqueous DMSO binary mixtures: 3: DMSO, 4: 1-methyl-2-pyrrolidone; NADES (0.8) + water (0.2) formulations with 5: glycerol, 6: (3S)-oxolan-3-ol, 7: (3R,4S)-oxolane-3,4-diol, 8: (1R,2S)-cyclobut-3-ene-1,2-diol, 9: 2,3-dihydroxycyclopropan-1-one, 10: 2,3-dihydroxycyclopentan-1-one, 11: 1-(hydroxymethyl) cycloprop-2-en-1-ol, 12: 2,2-dihydroxycyclopropan-1-one.

The purpose of the theoretical part of this paper is not merely the formulation of a theoretical model enabling accurate back computations of theophylline solubility but also the development of a reliable and extendable approach applicable for screening of new candidates for promising solubility enhancers. Bearing in mind that any extrapolation based on a single model cannot guarantee the expected reliability, even if back-computations match the fitted data set very closely, the ensemble of SANNs was developed, and the averaged values of predicted solubility were used for final screening purposes. Hence, the sets of 150 neat solvents, 250 binary mixtures and 1500 NADES were analysed, for which theophylline solubility was computed using the developed model. It is fair to say that not all values obtained during this stage fulfil the formal restrictions, and occasionally (less than 0.4%), the computed solubility values were outside of the meaningful physical range of mole fractions, and as such, they were discarded. That is why for every solvent, theophylline solubility was averaged over all SANNs included in the ensemble. The necessity of this step is documented in Figure 7, where the values of predicted solubility were plotted as a function of the size of the ensemble formed by the systematic inclusion of an increasing number of SANNs sorted according to increasing values of their RMSD. Interestingly, the green-solid line plotted in Figure 7 representing theophylline solubility prediction in DMSO is almost constant. This proves that the solubility values back-computed using different SANNs ensembles are stable and match the experimental ones. Among considered neat solvents, it was found that theophylline has the highest affinity toward solvents of class IV, grouping aprotic highly dipolar solvents. It is worth mentioning that DMSO is also classified as this type of solvent. The highest predicted solubility in neat solvents was found for 1-methyl-2-pyrrolidone (NMP), suggesting slightly higher efficiency compared to DMSO. However, it is worth emphasising that the computed solubility value was quite sensitive to the size of the SANNs ensemble. As it was presented in Figure 7 by a green-dashed line, the predicted value of theophylline solubility in NMP exhibits quite slow convergence, and prediction stabilises after as many as 35 networks used for averaging. Besides, some representatives of class II, i.e., weak electron-pair donor bases, such as morpholine and sulfolane, reached a solubility level comparable to DMSO. Meanwhile, m-cresol belonging to class IX of organic acidic compounds was identified as a solvent as effective as DMSO. Furthermore, screening of binary mixtures was also quite efficient, and one binary composition was found with potentially higher solubility compared to the DMSO-water mixture. It is quite interesting to note that the NMP-water mixture was identified as a promising candidate for solubility enhancement, which is consistent with predictions for neat solvents. No other binary mixture offering higher solubility was found. The last screening was devoted to finding NADES components more effective than glycerol. As it is documented in Figure 7, this step was also successful, and several compounds were identified as potentially more efficient solubilisers. Among all considered candidates, two tetrahydrofuran analogues (3-hydroxytetrahydrofuran [CAS: 86087-23-2] and cis-tetrahydrofuran-3,4-Diol [CAS: 4358-64-9]) were found as the components of the most efficient NADES. Likewise, some hydroxy-analogues of cyclopropane and cyclopropene were identified as potentially interesting from the perspective of an optimisation of NADES compositions for enhancing theophylline solubility. The more detailed results of the screening are provided in Supplementary Materials, Figures S2–S4.

## 3. Materials and Methods

### 3.1. Materials

The theophylline (CAS: 58-55-9) considered in this study was obtained from Sigma-Aldrich (Poznań, Poland). The following organic solvents were used: dimethyl sulfoxide (CAS: 67-68-5), dimethylformamide (CAS: 68-12-2), 1,4-dioxane (CAS: 123-91-1), acetonitrile (CAS: 75-05-8), acetone (CAS: 67-64-1), methanol (CAS: 67-56-1), 1-propanol (CAS: 71-23-8), 1-butanol (CAS: 71-36-3), 1-pentanol (CAS: 71-41-0), 1-octanol (CAS: 111-87-5) and ethyl acetate (CAS: 141-78-6); all of which were reagent grade and purchased from Avantor Performance Materials, Gliwice, Poland. Natural deep eutectic solvents constituents included: choline chloride (CAS: 67-48-1), glucose (CAS: 50-99-7), fructose (CAS: 57-48-7), sorbitol (CAS: 50-70-4), xylitol (CAS: 87-99-0), maltose (CAS: 69-79-5), saccharose (CAS: 57-50-1) and glycerol (CAS: 56-81-5), all supplied by Sigma-Aldrich.

### 3.2. Preparation of Calibration Curve

A stock solution of theophylline with a concentration of 1.6 mg/mL in methanol was prepared in a 100 mL volumetric flask. From this solution, successively smaller amounts were taken and diluted with methanol in 10 mL volumetric flasks, which led to solutions with decreasing concentrations of theophylline. Solutions prepared in this manner were measured spectrophotometrically, which enabled for obtaining a relationship between the concentration of theophylline in solution and the obtained absorbance values, measured at 270 nm. Three separate curves were obtained, and their average was used as the final calibration curve for theophylline determination. The determination coefficient R^2^, as well as the limits of detection (LOD) and quantification (LOQ), were also calculated. The detailed values of concentration and absorbance, as well as the obtained parameters of the curve, can be found in Supplementary Materials Table S1a,b, respectively.

### 3.3. Preparation of Samples in Organic Solvents and Their Binary Mixtures with Water

Solvent mixtures were prepared by mixing the appropriate amounts of organic solvent and water in 10 mL volumetric flasks in order to obtain different molar ratios. Further, test tubes were filled with an excess amount of theophylline for ensuring that a saturated solution is obtained. To these test tubes, the prepared solvent mixture or a pure solvent was added. Three samples were prepared for each tested solvent, both neat and binary. The samples were placed in an Orbital Shaker Incubator ES-20/60 from Biosan (Riga, Latvia) and incubated for 24 h at 25 °C. The temperature setting accuracy was 0.1 °C, and the daily deviations of temperature were at a level of ±0.5 °C. The mixing of solutions was assured by their shaking at 60 rev/min. After incubation, the samples were filtered in a multi-step procedure with the use of a PTFE syringe filter with 0.22 μm pore size. All of the test tubes, syringes, filters, etc., were pre-heated in the same incubator in order to obtain the same temperature as the sample. From the obtained filtrate, a fixed amount of solution was transferred to other test tubes containing methanol as a diluting agent and later measured spectrophotometrically. Meanwhile, the density of the solution was measured in 10 mL volumetric flasks and used for determining the mole fractions of theophylline in the solution.

### 3.4. Preparation of Samples in NADES and Their Binary Mixtures with Water

Natural deep eutectic solvents (NADES) were prepared by mixing choline chloride and the second constituent in 1:1 molar ratios. Mixtures prepared in this way were placed in sealed test tubes and put in a water bath at 90 °C until a uniform solution was formed. NADES were used in their neat form and as cosolvents mixed with water in different molar ratios. An excess amount of theophylline was then added to the NADES solution, and the samples were incubated for 24 h at 25 °C, as was the case for organic solvents. Because of the increased viscosity and density, the samples were centrifuged (1000 rev/min for 5 min) using a Hettich EBA (Tuttlingen, Germany) 20 centrifuge so that the undissolved precipitate remained on the bottom of the test tube. The samples were then filtered, and their absorbances were measured after dilution, as described earlier.

### 3.5. Solubility Measurements

The concentration of theophylline in the samples was determined spectrophotometrically with the use of an A360 spectrophotometer from AOE Instruments (Shanghai, China). The measurements were carried out in a wavelength range from 190 nm to 700 nm with a resolution of 1 nm. Before the actual measurements, the instrument was calibrated using methanol as a reference. The whole spectra were registered for confirming that the position of the maximum was not shifted after solubility measurements. In order to ensure that the absorbance values are not outside the linearity limit, several dilutions of the samples using methanol were made, depending on the concentration of theophylline in the samples. Based on the slope coefficient and y-intercept of the calibration curve and the absorbance values measured at 270 nm, the concentration of theophylline in the samples was determined. Three samples were measured for each system, and their mean concentrations were determined and expressed as mole fractions together with the standard deviation values.

### 3.6. Differential Scanning Calorimetry Measurements 

In order to characterise the pseudo-polymorphic behaviour of theophylline in the presence of water and organic solvents, differential scanning calorimetry (DSC) measurements were utilised for co-ground samples. The mechanochemical approach was selected due to its efficiency and previously documented application of co-grinding in successful theophylline hydrate synthesis [97].

Theophylline samples (0.4 g) were co-ground in a ball mill with appropriate amounts of pure water, water-methanol mixture and pure methanol. This step was performed using a Retsch MM 200 mill, Haan, Germany (25 Hz, 30 min.). The samples were ground in 5 mL stainless steel jars with two stainless steel balls. Then, the obtained powders were analysed using the DSC method. For this purpose, the DSC 6000 (PerkinElmer, Waltham, MA, USA) calorimeter was applied. The heating rate was set to 10 K/min. The inert atmosphere was provided by nitrogen flow (5.0 grade, Linde, 20 mL/min). The samples were measured in standard aluminium pans. Prior to the measurements, the calorimeter was calibrated using indium and zinc melting standards provided by the DSC manufacturer (PerkinElmer). 

### 3.7. COSMO-RS Solubility Computations

The amount of solute dissolved at a given temperature in any solvent is governed by the thermodynamics of the solid-liquid phase equilibrium, in which the value of the chemical potential of the i-th solute in its saturated state is identical to the value of the chemical potential of the pure solid phase:

(1) $$\mu_{i}^{s}=\mu_{i}^{liq}$$

(2) $$\mu_{i}^{o,s}+RTln\left(a_{i}^{s}\right)=\mu_{i}^{o,liq}+RTln\left(\gamma_{i}^{sat}x_{i}^{sat}\right)$$

where $\gamma_{i}^{sat}$ is the activity coefficient of the solute in the saturated solution of solute concentration equal to $x_{i}^{sat}$; T and R represent the temperature and the gas constant, respectively. For practical applications, the above equation requires the definition of the thermodynamic reference states, $\mu_{i}^{o,s}$ and $\mu_{i}^{o,liq}$. Here a commonly accepted convention is assumed [104] in which the reference state for the solid phase is the solid itself, and consequently, the activity of the solid equals the activity of the solute, *a^s^*, in the saturated solution. The thermodynamic reference state for the solute in the solution is defined as a pure compound supercooled melt at a given temperature corresponding to the conditions of the solubility measurements, $a_{i}^{sat}=\gamma_{i}^{sat}x_{i}^{sat}$. The activity of the solid phase depends on the fusion properties:

(3) $$ln\left(a_{i}^{s}\right)=ln\left(\gamma_{i}^{sat}x_{i}^{sat}\right)=-\frac{\Delta_{fus}{\bar{G}}_{i}^{m}}{RT}$$

where $\Delta_{fus}{\bar{G}}_{i}^{m}$ is the partial molar Gibbs energy of melting at the solubility measurements conditions, which by definition is equal to zero for the pure solute at its melting point. The COSMO-RS is a theory [64,105,106] of the liquid state and, as such, offers a direct calculation of chemical potentials of components in the bulk phase by calculating molecular interactions as local pair interactions of segments of molecular COSMO-surfaces. Practical solubility computations within the COSMO-RS approach are done by iteratively solving Equation (3), assuming that the values of Gibbs free energy of fusion are provided from external sources. Hence, the working equation has the following form:

(4) $$ln\left(\gamma_{i}^{sat,i+1}x_{i}^{sat,i+1}\right)=\frac{1}{RT}\left(\mu_{i}^{o,liq}-\mu_{i}^{\left(i\right)}\left(\gamma_{i}^{sat,i}x_{i}^{sat,i}\right)+\max\left(0,\Delta_{fus}{\bar{G}}_{i}^{m}\right)\right)$$

In the above equation, superscripts *i* and *i* + 1 denote the values obtained in two subsequent iterations. The iterative cycle is repeated until convergence is achieved, which means that the computation is supposed to be completed if the difference in the computed solubility drops below a defined threshold value. 

The quantum chemical part of the COSMO calculations provides information regarding the interactions of discrete polarisation change densities of the contacting surface segments representing a molecule embedded in a virtual conductor. Such microscopic state properties are related to macroscopic thermodynamic properties of a liquid by statistical thermodynamics through the analysis of density probability distributions termed σ-profiles and σ-potentials. Integration of the latter over the surface leads to the residual contribution to the chemical potential allowing for predictions of almost all thermodynamic properties, including activity coefficients, excess properties or partition coefficients and solubility:

(5) $$\mu_{s}\left(\sigma \right)=-\frac{RT}{a_{aff}}ln\left[\int P_{s}\left(\sigma^{\prime }\right)exp\left\{\frac{a_{aff}}{RT}\left[\mu_{s}\left(\sigma^{\prime }\right)-E_{misfit}\left(\sigma ,\sigma^{\prime }\right)-E_{HB}\left(\sigma ,\sigma^{\prime }\right)\right]\right\}d\sigma^{\prime }\right]$$

The actual calculations of these properties require a proper representation of the molecular structure. This is typically done by an adequate exploration of the conformational space using COMSOconf for generating the most energetically favourable structures. All computations were performed with the aid of TURBOMOLE rev. V7.5.1, Karlsruhe, Germany interfaced with TmoleX 2021 (version 21.0.1, BIOVIA, San Diego, CA, USA) using RI-DFT BP86 (B88-VWN-P86) functional and def-TZVP basis set for geometry optimisation and def2-TZVPD basis set for single-point calculations with the inclusion of the fine grid tetrahedron cavity and the inclusion of parameter sets with hydrogen bond interaction and van der Waals dispersion term based on the “D3” method of Grimme et al. [107]. All of the solubility calculations were performed using COSMOtherm (version 20.0.0, revision 5273M, BIOVIA, San Diego, CA, USA) [108] with BP_TZVPD_FINE_20.ctd parametrization.

### 3.8. Molecular Descriptors

According to COSMO-RS theory, any bulk system is modelled as an ensemble of closely packed ideally screened molecules enclosed by a virtual conductor. The origin of Coulomb interactions comes from the screening of electrostatics of two different contacting segments by their surroundings and the back-polarisation of the solute molecule. The specific interaction energy per unit area resulting from this “misfit” stands for the electrostatic contribution to the total energy:

(6) $$E_{MF}\left(\sigma ,\sigma^{\prime }\right)=a_{eff}\frac{a^{\prime }}{2}{\left(\sigma +\sigma^{\prime }\right)}^{2}$$

where *a_eff_* is the effective contact area between two surface segments and α′ is an adjustable parameter. Furthermore, the hydrogen bonding (H-Bond) is similarly described by screening the charge density of the two adjacent centres of strongly negative and positive densities of donors and acceptors, respectively. It is assumed that such interactions take place if two sufficiently polar pieces of the surface of opposite polarity are in contact and defined by a mathematical formula in the following form:

(7) $$E_{HB}\left(\sigma ,\sigma^{\prime }\right)=a_{eff}c_{HB}\min\left(0;\min\left(0;\sigma_{don}+\sigma_{HB}\right);\min\left(0;\sigma_{acc}-\sigma_{HB}\right)\right)$$

where σ*HB* and σ′*HB* are adjustable parameters. Additionally, the formalism of COSMO-RS also takes into account Van der Waals (VdW) interactions between surface segments. Such energetic contributions are defined via:

(8) $$E_{vdW}\left(\sigma ,\sigma^{\prime }\right)=a_{rff}c_{vdW}\left(\sigma ,\sigma^{\prime }\right)=a_{eff}\left(\tau_{vdW}+\tau_{\prime vdW}\right)$$

where *τvdW*, *τvdW*′, and *cvdW* are element specific adjustable parameters. The vdW energy is dependent only on the element type of the atoms that are involved in surface contact. Since any prediction applied within the COMO-RS framework generate these energetic contributions, they are directly available from outputs of different properties, including solubility. Hence, the energetic information is taken as a set of universal descriptors characterising the properties of any system. For practical purposes, the values of misfit ($E_{i}^{misfit}$), H-Bond $\left(E_{i}^{HB}\right)$ and van der Waals ($E_{i}^{VdW}$) mean interaction energies computed for the pure i-th components were augmented with a set of relative values defined as follows:

(9) $$e_{i}^{j}=\frac{E_{i}^{j}}{E_{i}^{misfit}+E_{i}^{HB}+E_{i}^{VdW}}$$

where *j* stands for misfit, HB or VdW contribution. In the case of multicomponent systems, the descriptor values were computed as a weighted sum with molar fraction.

### 3.9. Machine Learning Protocol

The non-linear modelling was used for the development of an ensemble of artificial neural networks. The flexibility of computations offered by STATISTICA software (version 13, TIBCO Software Inc., Palo Alto, CA, USA) was used for Statistica Automated Neural Networks (SANNs) growth. Simple one-layer infrastructure was assumed. The input layer comprised the sets of seven molecular descriptors depicting intermolecular interactions, and the only expected value in the output layer was the estimated solubility. The default settings of SANN generation were assumed, allowing for the automatic selection of initial and output neurons to be in the functional form of linear, logistic, Tanh, exponential or sin functions. Only algorithms utilising multilayer perceptrons (MLP) were allowed during the machine learning phase. The model was constructed using 160 points, and the dataset was automatically split into training (70%), test (15%) and validation (15%) subsets. The sum-squares error function (SOS) was used as an error function for the training of the neural networks. The ensemble of networks was constructed by a successful accumulation of networks fulfilling two conditions, namely RMDS < 0.04 and the largest number of outliers not exceeding four cases. The applicability domain of each model was computed using the hat value according to standard methodology [109,110,111].

### 3.10. Solvents Selection for Solubility Screening

The list of neat solvents was taken from the literature [112,113], where classification into ten chemically intuitive classes was suggested. Hence, the screening of this set of neat solvents might provide clues about solvent types offering higher solubility of theophylline. The second group of solvents was taken from an in house database of binary solvents effectively used for solubility measurements. In principle, binary mixtures might be constructed by all possible combinations of neat solvents, but this is not recommended since it is not obvious which pairs of solvents are miscible. Hence, the list was restricted only to such mixtures, which were already utilised as solvents for solubility measurements. The last set was formulated using replacers for glycerol in NADES formulations with choline chloride. For this purpose, the PubChem database was searched for analogues of glycerol and other sugars used in this study. After removing duplicates, the initial list was inspected for systems containing atoms other than C, H and O, since the search was narrowed only to polyol analogues. These were removed from the list along with radicals, anions and isotopomers. Finally, 1050 compounds were used for the NADES screening in compositions mimicking prescriptions of real NADES systems. For every solvent, the values of molecular descriptors were computed and used for theophylline solubility computations with an aid to the developed SANNs ensemble and averaged.

## 4. Conclusions

Solubility of theophylline is quite poor in water and the majority of neat organic solvents. The rare exception is DMSO, which as an aprotic highly dipolar solvent, is commonly used for the solubility enhancement of many APIs. Likewise, a serious solubility gain is often observed for binary mixtures of some organic solvents with water. These observations are intuitive and agree with expectations arising from chemical experiences. However, the performed experiments provided additional less obvious characteristics. Namely, a non-trivial synergistic effect was measured for aqueous solutions with five different organic solvents. In each case, the highest solubility was found to be corresponding to a unimolar proportion of organic solvent and water. The 1:1 mixture of DMSO with water is characterised by 1.27 times higher solubility compared to pure DMSO. A similar effect was observed for mixtures of such organic solvents like DMF, 1,4-dioxane and some aliphatic alcohols. For further improvements of theophylline dissolution, several NADES compositions were tested, and many of them not only offer better solubility at room temperature but also exhibit an even stronger synergistic effect. The unimolar mixtures of choline chloride with glycerol and other polyols have been found as efficient solubilisers preserving solubility enhancement after dilution with water. The solubility measurements revealed that the most effective designed solvent comprises a unimolar composition of choline chloride with glycerol in an aqueous formulation comprising 0.2 molar fractions of water. It is quite interesting to see that the water dilution of NADES promotes the solubility of theophylline since typical NADES possess very strong hygroscopic properties and their physicochemical properties are often very sensitive to the amount of contained water. Here it was proved that for solubility enhancement of theophylline, the hygroscopicity of NADES is not a constrain but rather promotes hydrotropic features.

The experimental screening of theophylline solubility was extended with theoretical analysis using non-linear modelling, which resulted in an ensemble of SANNs enabling for very accurate mole fraction back-computations and also for extensive screening of neat solvents, their binary mixtures and a variety of polyols in NADES formulations containing choline chloride. Among 153 neat solvents grouped into ten classes, the solvents of the first choice are aprotic highly dipolar solvents and such ones as NMP, morpholine and sulfolane are alternatives to DMSO. This conclusion also holds for aqueous binary formulations as inferred from the screening of theophylline solubility in 252 binary solvent mixtures. The model formulated based on the neural networks ensemble was also effective in the optimisation of NADES constituents. Several replacers of glycerol were found offering significant solubility enhancement, among which hydroxy-analogues of tetrahydrofuran, cyclopropane and cyclopropene were potentially more efficient NADES than the one with glycerol. Future experiments for theophylline solubility enhancement might be directed by these observations.

Additionally, the performed non-linear analysis also provides methodological clues for the further development of accurate models for solubility predictions. It is not advised to use a single neural network even if it offers high accuracy in back computed values and matches to the experimental ones. Occasionally, even for systems within the applicability domain of the model, the predicted values do not guarantee mole fractions within the formal requirement belonging to the range between zero and unity. This problem is not very serious since it has been encountered in less than 0.4% of cases. A more severe problem is posed by the occasional inconsistencies between predictions of different SANNs, even with comparable RMSDs. Fortunately, taking into account a sufficiently extended ensemble of neural networks guarantees the convergence of predicted solubility values provided that each individual network is satisfactorily accurate. Although depending on the considered solvent, the size of ensemble necessary for convergence varies, using at least 30 networks was found to be generally sufficient. From the perspective of practical implementations, this is not a serious restriction since each step can be easily automated.

## Data Availability

All data supporting reported results are available on request from the corresponding author.

## Supplementary Materials

The following are available online at https://www.mdpi.com/article/10.3390/ijms22147347/s1.
