# Supplementary material for: Solvent Screening for Solubility Enhancement of Theophylline in Neat, Binary and Ternary NADES Solvents: New Measurements and Ensemble Machine Learning
Source: Int J Mol Sci. 2021 Jul 8;22(14):7347. doi: 10.3390/ijms22147347 (PMC8304713; doi:10.3390/ijms22147347)
Supplement: Supplementary file 1 [file ijms-22-07347-s001.zip › ijms-1243634-supplementary.pdf]

Solvent screening for solubility enhancement of Theophylline in neat, binary and ternary NADES solvents: new measurements and ensemble machine learning.

Piotr Cysewski[1,*], Patryk Cymerman[1], Tomasz Jeliński[1] and Maciej Przybyłek[1]

[1] Department of Physical Chemistry, Faculty of Pharmacy, Collegium Medicum of Bydgoszcz, Nicolaus Copernicus University in Toruń, Kurpińskiego 5, 85-950 Bydgoszcz, Poland
* Correspondence: piotrc@cm.umk.pl

Supplementary materials

List of figures and tables

Table S1a. Concentrations of Theophylline solutions and corresponding mean absorbance and standard deviation values, used during preparation of the calibration curve.
Table S1b. Parameters of the obtained calibration curve for Theophylline solubility determination.
Table S2. Comparison of Theophylline solubility at 25°C obtained in this study and the results taken from literature.
Table S3. Mole fractions ($\cdot 10^4$) and standard deviation values ($\cdot 10^4$) of Theophylline at 25°C in neat organic solvents and water.
Table S4. Mole fractions ($\cdot 10^4$) and standard deviation values ($\cdot 10^4$) of Theophylline at 25°C in binary solvents comprising water and an organic solvent in different proportions.
Table S5. Mole fractions ($\cdot 10^4$) and standard deviation values ($\cdot 10^4$) of Theophylline at 25°C in pure Natural Deep Eutectic Solvents.
Table S6. Mole fractions ($\cdot 10^4$) and standard deviation values ($\cdot 10^4$) of Theophylline at 25°C in mixture containing water and Natural Deep Eutectic Solvents in different proportions.
Table S7
Characteristics of clusters of developed artificial networks used for Theophylline solubility predictions in neat solvents, binary aqueous mixtures and ternary NADES choline chloride based solvents.
Figure S1.
The Importance of molecular descriptors in the development of SANNs. Red bold line represents the mean value, while open circles characterize the most efficient network (SANN1).
Figure S2.
(top) The results of screening of Theophylline solubility in neat solvents using the cluster of 30 neural networks. For comparison purposes the experimental solubility in DMSO was provided. The very first point corresponds to predications of the most accurate network SANN1.
(bottom) Distributions of hat values.
Figure S3.
(top) The results of screening of Theophylline solubility in 228 binary solvents mixtures using the cluster of 30 neural networks. For comparison purposes the experimental solubility in 1:1 [DMSO+water] solution was provided. Networks are numbered according to decreasing accuracy (RMSD values). (bottom) Distributions of hat values.
Figure S4.
(top) The results of screening of Theophylline solubility in aqueous NADES formed by 1050 glycerol replacers in condition mimicking experimental conditions. For comparison purposes the experimental solubility in 0.8:0.2 [ChCl+glycerol]:[water] solution was provided. (bottom) Distributions of hat values.

Table S1a. Concentrations of Theophylline solutions and corresponding mean absorbance and standard deviation values, used during preparation of the calibration curve.

C [mg/ml]	A_{mean}	SD
0.01800	1.271	0.023
0.01440	1.012	0.012
0.01200	0.859	0.016
0.01029	0.730	0.023
0.00900	0.633	0.021
0.00800	0.548	0.023
0.00720	0.513	0.005
0.00655	0.457	0.014
0.00600	0.421	0.007
0.00554	0.392	0.005
0.00514	0.369	0.009
0.00480	0.344	0.010

Table S1b. Parameters of the obtained calibration curve for Theophylline solubility determination.

parameter	value
a	70.595
b	$-1.11 \cdot 10^{-16}$
R^2	0.9993
LOD	$3.56 \cdot 10^{-4}$ mg/ml
LOQ	$1.07 \cdot 10^{-3}$ mg/ml

Table S2. Comparison of Theophylline solubility at 25°C obtained in this study and the results taken from literature.

solvent	x_T [$\cdot 10^4$]		relative difference
	literature	results	
water[a]	6.20	6.10	-1.53%
water[b]	6.30	6.10	-3.10%
methanol[a]	13.70	13.64	-0.41%
methanol[b]	13.30	13.64	2.58%
1-propanol[a]	13.58	13.39	-1.38%
ethyl acetate[a]	6.73	6.89	2.39%
acetone[a]	9.30	9.51	2.22%

[a]Zhong et al. [95] [b]Cárdenas et al. [96]

Table S3. Mole fractions (·10^4) and standard deviation values (·10^4) of Theophylline at 25°C in neat organic solvents and water.

solvent	$x_T \cdot 10^4$	SD·10^4
**DMSO**	70.960	0.283
**DMF**	59.210	0.431
**1,4-dioxane**	31.669	0.582
**acetone**	9.532	0.241
**acetonitrile**	6.779	0.130
**1-propanol**	13.393	0.125
**1-butanol**	10.229	0.112
**1-pentanol**	6.879	0.144
**1-octanol**	7.270	0.105
**ethyl acetate**	6.891	0.060
**methanol**	13.644	0.041
**water**	6.103	0.123

Table S4. Mole fractions (·10^4) and standard deviation values (·10^4) of Theophylline at 25°C in binary solvents comprising water and an organic solvent in different proportions.

$x_{solvent}$	DMSO		DMF		1,4-dioxane		1-butanol		1-propanol	
	$x_T \cdot 10^4$	SD·10^4	$x_T \cdot 10^4$	SD·10^4	$x_T \cdot 10^4$	SD·10^4	$x_T \cdot 10^4$	SD·10^4	$x_T \cdot 10^4$	SD·10^4
**0.00**	6.105	0.123	6.105	0.123	6.105	0.123	6.105	0.123	6.105	0.123
**0.03**	9.140	0.062	8.193	0.016	7.553	0.120	6.623	0.161	6.771	0.253
**0.07**	11.154	0.040	9.949	0.194	8.414	0.070	7.283	0.109	7.515	0.087
**0.10**	13.235	0.129	11.005	0.178	9.677	0.228	8.142	0.150	8.204	0.219
**0.13**	15.086	0.081	12.968	0.134	11.076	0.135	9.363	0.201	9.234	0.187
**0.17**	18.092	0.092	16.193	0.149	13.108	0.020	11.243	0.249	10.785	0.236
**0.20**	21.915	0.148	19.924	0.248	15.114	0.129	13.262	0.108	12.600	0.193
**0.32**	42.102	0.259	34.769	0.577	29.278	0.457	22.076	0.623	20.655	0.397
**0.50**	90.130	0.560	76.421	0.583	62.371	0.448	43.466	0.221	35.030	0.084
**0.68**	83.259	0.827	70.633	0.538	52.440	0.747	27.304	0.131	23.945	1.568
**0.80**	77.514	0.577	64.674	0.730	40.175	0.520	18.180	0.571	16.323	0.245
**1.00**	70.960	0.283	59.210	0.431	31.669	0.582	10.229	0.112	13.393	0.125

Table S5. Mole fractions (·10^4) and standard deviation values (·10^4) of Theophylline at 25°C in pure Natural Deep Eutectic Solvents.

NADES constituent	$x_T \cdot 10^4$	SD·10^4
**glycerol**	128.174	0.682
**fructose**	67.792	0.723
**glucose**	73.447	0.755
**sorbitol**	103.631	1.200
**xylitol**	92.610	0.938
**saccharose**	43.155	1.011
**maltose**	37.956	0.951

Table S6. Mole fractions (·10[4]) and standard deviation values (·10[4]) of Theophylline at 25°C in mixture containing water and Natural Deep Eutectic Solvents in different proportions.

x_{NADES}	ChCl+glycerol		ChCl+sorbitol		ChCl+xylitol		ChCl+glucose	
	$x_T \cdot 10^4$	$SD \cdot 10^4$	$x_T \cdot 10^4$	$SD \cdot 10^4$	$x_T \cdot 10^4$	$SD \cdot 10^4$	$x_T \cdot 10^4$	$SD \cdot 10^4$
**0.00**	6.105	0.123	6.105	0.123	6.105	0.123	6.105	0.123
**0.03**	6.454	0.069	6.387	0.012	6.221	0.036	6.413	0.027
**0.07**	7.145	0.068	6.799	0.066	6.560	0.047	6.852	0.022
**0.10**	8.021	0.155	7.179	0.054	6.982	0.048	7.603	0.061
**0.13**	9.116	0.110	7.907	0.121	7.602	0.046	8.663	0.036
**0.17**	10.894	0.062	8.976	0.074	8.481	0.042	10.089	0.025
**0.20**	13.517	0.119	10.986	0.074	10.395	0.082	12.601	0.068
**0.32**	27.081	0.302	23.009	0.275	18.977	0.199	23.011	0.076
**0.50**	56.226	0.371	64.337	0.374	53.438	0.122	48.872	0.310
**0.68**	123.123	1.116	115.068	0.390	101.261	0.421	82.987	0.675
**0.80**	146.569	0.718	123.827	0.699	113.936	0.313	93.764	0.958
**1.00**	128.174	0.682	103.813	0.914	92.610	0.938	73.357	0.082

Table S7. Characteristics of clusters of developed artificial networks used for Theophylline solubility predictions in neat solvents, binary aqueous mixtures and ternary NADES choline chloride based solvents.

SANN code	neurons	outliers	RMSD $\cdot 10^3$	Error function	Activation function	Output function	Trainig algorithm
SANN1	**10**	**1**	**29.3**	**SOS**	**Tanh**	**Tanh**	**BFGS 341**
SANN2	8	2	33.3	SOS	Tanh	Linear	BFGS 400
SANN3	12	1	33.6	SOS	Tanh	Linear	BFGS 339
SANN4	9	3	33.8	SOS	Tanh	Tanh	BFGS 278
SANN5	11	2	34.2	SOS	Tanh	Tanh	BFGS 364
SANN6	11	2	34.4	SOS	Tanh	Linear	BFGS 228
SANN7	12	3	34.5	SOS	Tanh	Tanh	BFGS 447
SANN8	12	2	35.1	SOS	Tanh	Linear	BFGS 333
SANN9	6	2	35.2	SOS	Tanh	Linear	BFGS 357
SANN10	9	3	35.4	SOS	Tanh	Linear	BFGS 279
SANN11	11	2	36.9	SOS	Tanh	Tanh	BFGS 289
SANN12	10	2	36.9	SOS	Tanh	Linear	BFGS 209
SANN13	9	4	37.1	SOS	Logistic	Linear	BFGS 408
SANN14	12	2	37.3	SOS	Tanh	Tanh	BFGS 227
SANN15	11	4	37.3	SOS	Tanh	Tanh	BFGS 273
SANN16	12	4	37.5	SOS	Tanh	Tanh	BFGS 348
SANN17	12	2	37.5	SOS	Tanh	Linear	BFGS 260
SANN18	8	4	37.6	SOS	Tanh	Tanh	BFGS 314
SANN19	11	4	37.7	SOS	Tanh	Linear	BFGS 281
SANN20	8	1	37.9	SOS	Tanh	Linear	BFGS 226
SANN21	12	2	37.9	SOS	Tanh	Linear	BFGS 217
SANN22	10	3	38.2	SOS	Tanh	Linear	BFGS 181
SANN23	12	1	38.2	SOS	Tanh	Exponential	BFGS 309
SANN24	12	4	38.2	SOS	Tanh	Tanh	BFGS 203
SANN25	11	2	38.3	SOS	Tanh	Linear	BFGS 273
SANN26	11	4	38.5	SOS	Tanh	Tanh	BFGS 259
SANN27	9	4	38.6	SOS	Logistic	Linear	BFGS 299
SANN28	6	4	38.7	SOS	Logistic	Tanh	BFGS 338
SANN29	12	2	38.8	SOS	Exponential	Exponential	BFGS 293
SANN30	10	3	38.9	SOS	Exponential	Exponential	BFGS 278
SANN31	10	3	39.0	SOS	Tanh	Exponential	BFGS 410
SANN32	12	1	39.0	SOS	Exponential	Exponential	BFGS 374
SANN33	7	2	39.2	SOS	Tanh	Tanh	BFGS 325
SANN34	11	1	39.3	SOS	Exponential	Exponential	BFGS 303
SANN35	8	3	39.6	SOS	Tanh	Tanh	BFGS 250
SANN36	11	2	39.6	SOS	Exponential	Exponential	BFGS 285
SANN37	12	3	39.9	SOS	Exponential	Logistic	BFGS 311
SANN38	12	3	39.9	SOS	Exponential	Exponential	BFGS 295
SANN39	10	3	39.9	SOS	Tanh	Linear	BFGS 268
SANN40	10	3	40.0	SOS	Tanh	Linear	BFGS 240

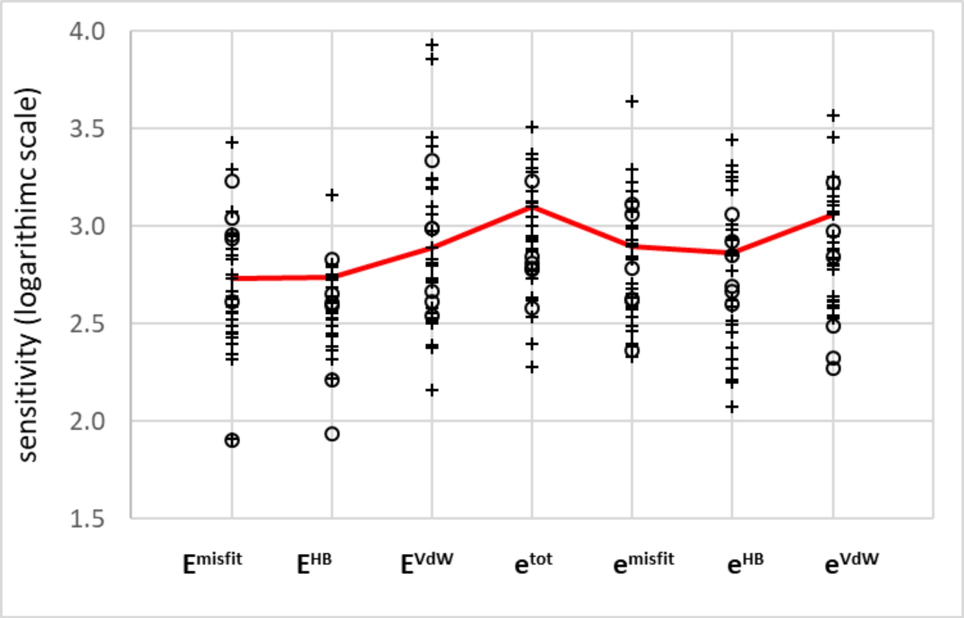

Figure S1. The Importance of molecular descriptors in the development of SANNs. Red bold line represents the mean value, while open circles characterize the most efficient network (SANN1).

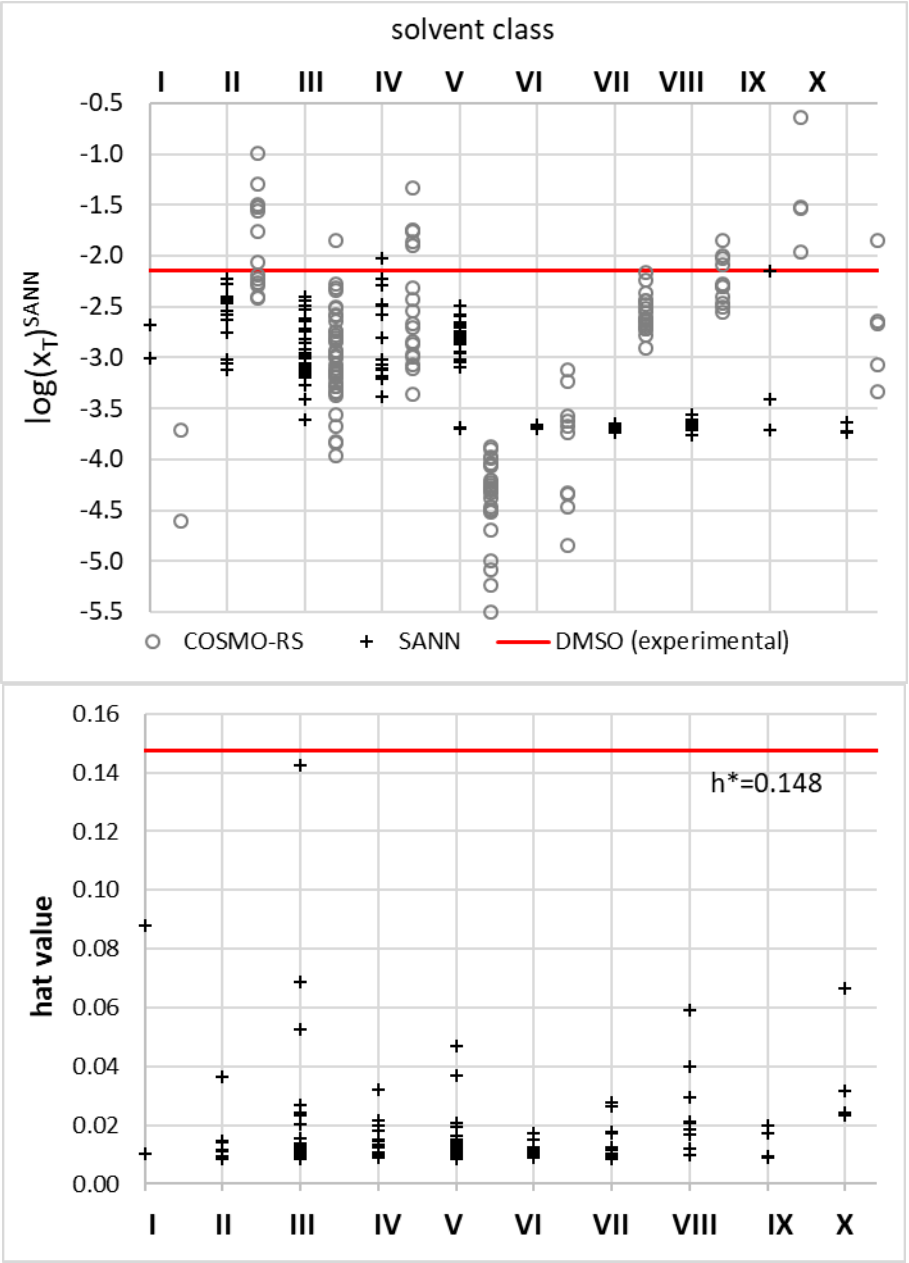

Figure S2.(top) The results of screening of Theophylline solubility in neat solvents using the cluster of 30 neural networks. For comparison purposes the experimental solubility in DMSO was provided. The very first point corresponds to predications of the most accurate network SANN1. The neat solvents classification (bottom) Distributions of hat values. The following classes were adopted according to Durand et al [102], namely I-strong electron pair, donor bases; II - weak electron pair, donor bases; III - aprotic dipolar; IV - aprotic highly dipolar; V – apolar; VI – asymmetric halogenated hydrocarbons; VII – amphiprotic; VIII -polar protic; IX - organic acidic compounds; X polar structured

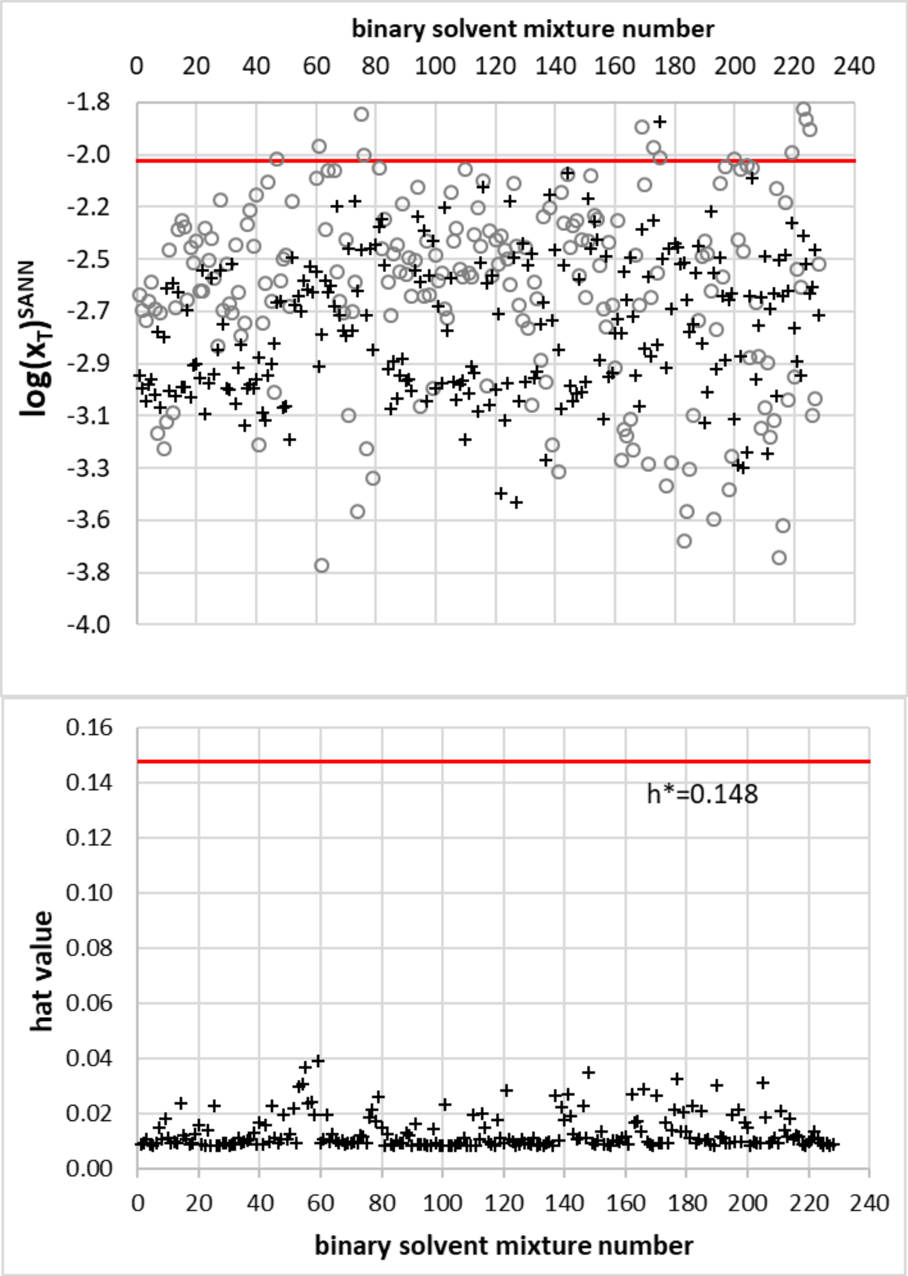

Figure S3. (top) The results of screening of Theophylline solubility in 228 binary solvents mixtures using the cluster of 30 neural networks. For comparison purposes the experimental solubility in 1:1 [DMSO+water] solution was provided. Networks are numbered according to decreasing accuracy (RMSD values). (bottom) Distributions of hat values.

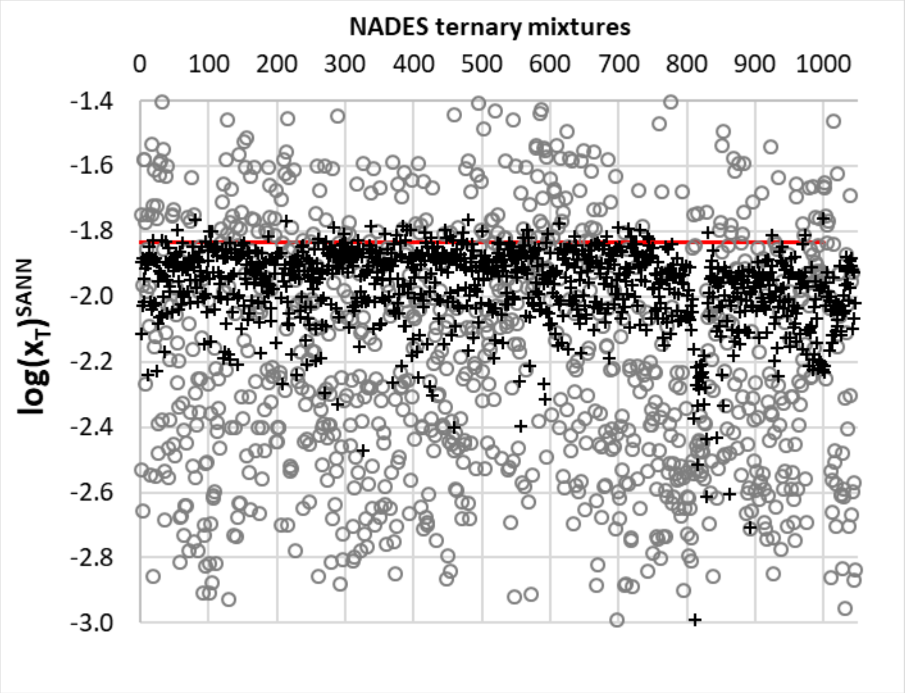

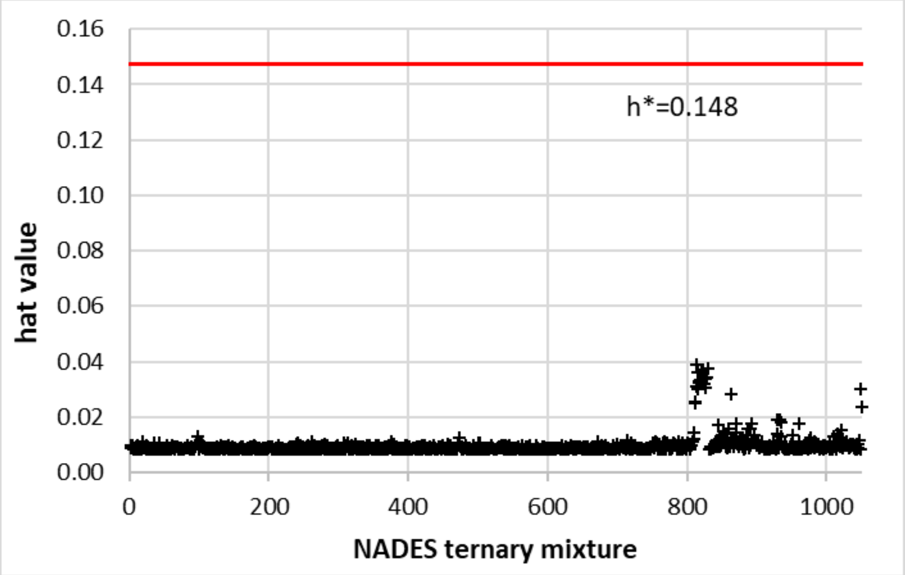

Figure S4. (top) The results of screening of Theophylline solubility in aqueous NADES formed by 1050 glycerol replacers in condition mimicking experimental conditions. For comparison purposes the experimental solubility in 0.8:0.2 [ChCl+glycerol]:[water] solution was provided. (bottom) Distributions of hat values.
